# Perturbation of transcriptome in non-neoplastic salivary gland epithelial cell lines derived from patients with primary Sjögren's syndrome

**DOI:** 10.1016/j.dib.2017.12.023

**Published:** 2017-12-12

**Authors:** Aigli G. Vakrakou, Alexandros Polyzos, Efstathia K. Kapsogeorgou, Dimitris Thanos, Menelaos N. Manoussakis

**Affiliations:** a Department of Pathophysiology, School of Medicine, National and Kapodistrian University of Athens, Athens, Greece; b Hellenic Pasteur Institute, Laboratory of Molecular Immunology, Athens, Greece; c Biomedical Research Foundation of the Academy of Athens, Athens, Greece; d Joint Academic Rheumatology Program, National and Kapodistrian University of Athens, Athens, Greece

## Abstract

The data presented here are related to the research article titled *“Impaired anti-inflammatory activity of PPARγ in the salivary epithelia of Sjögren's syndrome patients imposed by intrinsic NF-κB activation*” (Vakrakou et al., Journal of Autoimmunity, in press, 2017). In the cited manuscript, using comparative analyses of salivary gland biopsy specimens and ductal salivary gland epithelial cell (SGEC) lines from SS patients and disease controls, we have demonstrated that the ductal epithelia of SS patients display constitutively reduced PPARγ expression, transcriptional activity and anti-inflammatory function that were associated with cell-autonomously activated NF-κB and IL-1β pathways in these cells. Herein, the comparative transcriptome analysis of SGEC lines is presented. We show that the ductal epithelia of SS patients with severe lymphoepithelial lesions manifest constitutive perturbation in various inflammation- and metabolism related signaling pathways.

**Specifications Table**

**Table:** 

Subject area	Immunology, Rheumatology
More specific subject area	Transcriptome analysis of ductal salivary gland epithelial cell (SGEC) lines derived from patients with primary Sjögren’s syndrome (SS) and non-SS disease controls.
Type of data	Text file, graph and excel files.
How data was acquired	Affymetrix microarray technology (GeneChipHuGene 1.0ST arrays with 28,869 annotated genes) was used for the analysis of total gene expression profiles in SGEC lines. Microarray analysis was performed with the R statistical environment version 2.13 using the Bioconductor package. Gene ontology annotation of differentially expressed genes, signal pathway analysis and network construction was performed with the use of Ingenuity Pathway Analysis (IPA) software and the Kyoto Encyclopedia of Genes and Genomes (KEGG).
Data format	Analyzed
Experimental factors	No pretreatment of samples was performed.
Experimental features	Comparative total gene expression profiles in SGEC from controls and SS patients.
Data source location	Athens, Greece
Data accessibility	The data will be available with this article. All microarray data are available in the GEO archive under the accession number GSE97614.

**Value of the data**

• This is the first study to analyze the differential gene expression between non-neoplastic SGEC lines derived from SS patients and those from non-SS controls.
• The results of this study provide supportive evidence for the active role of ductal epithelia in the pathogenesis of SS, as well as for the occurrence of cell-autonomous aberrations in these cells.
• The identification of affected signaling pathways in the SGEC lines of SS patients sheds new light on the investigation of disease pathogenesis and for novel treatment targets.

## 1 Data

**Fig. 1:**
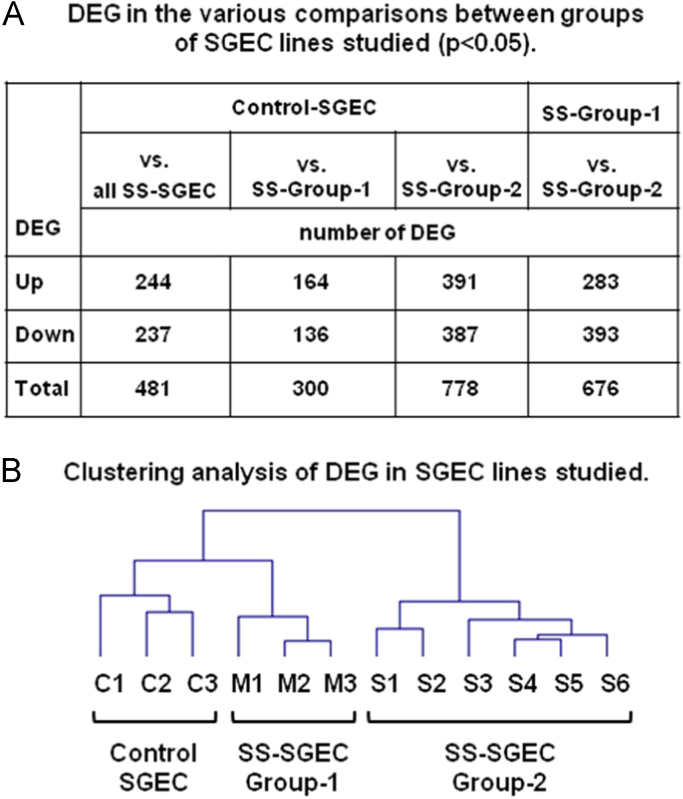
Transcriptome analysis reveals that SGEC lines derived from SS patients with severe histopathologic lesions are characterized by a distinct molecular signature. Microarray analysis of non-neoplastic SGEC lines derived from SS patients (SS-SGEC lines, *n* = 9) and non-SS controls (normal-SGEC lines, *n* = 3). Several differentially expressed genes (DEG; either up-regulated or down-regulated genes, for *p* < 0.05) were identified between the subgroups of SS-SGEC lines, namely the SS-Group-1 (*n* = 3; SGEC lines derived from SG biopsies with focus score < 2) and SS-Group-2 (n = 6; SGEC lines derived from SG biopsies with focus score ≥ 2) and the normal-SGEC lines. **A**. Numerical presentation of up-regulated and down-regulated DEG among the various group and subgroup comparisons (for *p* < 0.05). B. Dendrogram showing the clustering of DEG in control normal-SGEC lines (C1–C3), SS-Group-1 (M1–M3) and SS-Group-2 SGEC lines (S1–S6).

**Fig. 2:**
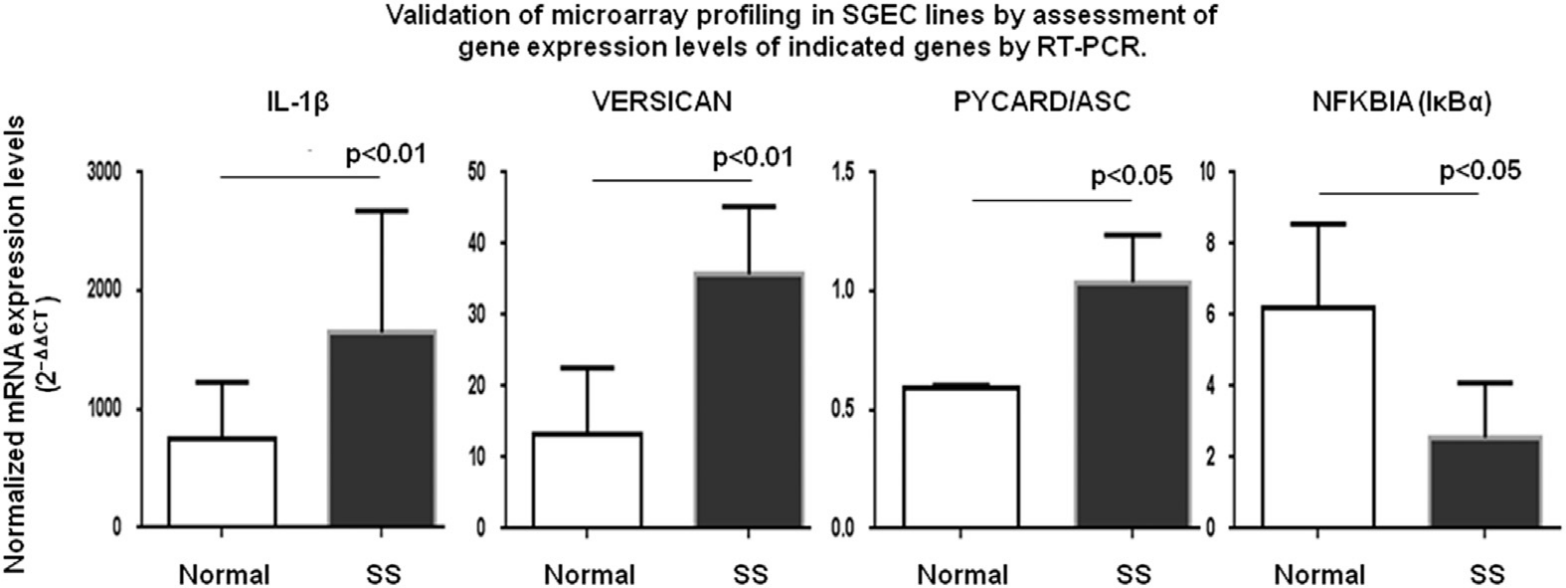
Validation of microarray analysis results. RT-PCR was performed to evaluate the mRNA expression levels of 4 genes found to be differentially expressed in SS-SGEC lines compared to normal-SGEC lines, according to the microarray analysis. The validation experiments were performed in 5 normal-SGEC (controls) and7 SS-SGEC lines (all belonging to SS-Group-2), which were different from those applied for microarray analysis. HPRT1 was included as an endogenous standard. The statistically-significant differences are shown. Group comparisons were performed by Mann-Whitney rank sum test.

**Fig. 3:**
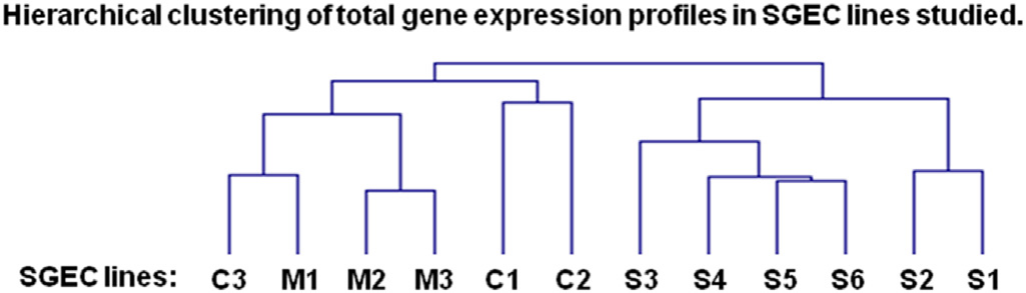
The hierarchical clustering of total gene expression profiles advocates for the distinctive biology of SGEC lines derived from SS patients with severe histopathologic lesions. Hierarchical clustering of total genes in array (uniquely represented 20.252 genes) was performed with the TMeV Software. For analysis of data, Pearson correlation co-efficiency and average linkage method were applied. C1–C3: normal-SGEC lines (controls), M1–M3: SS-Group-1 SGEC lines (derived from SG biopsies with focus score < 2), S1-S6: SS-Group-2 of SS-SGEC lines (derived from SG biopsies with focus score ≥ 2).

**Fig. 4:**
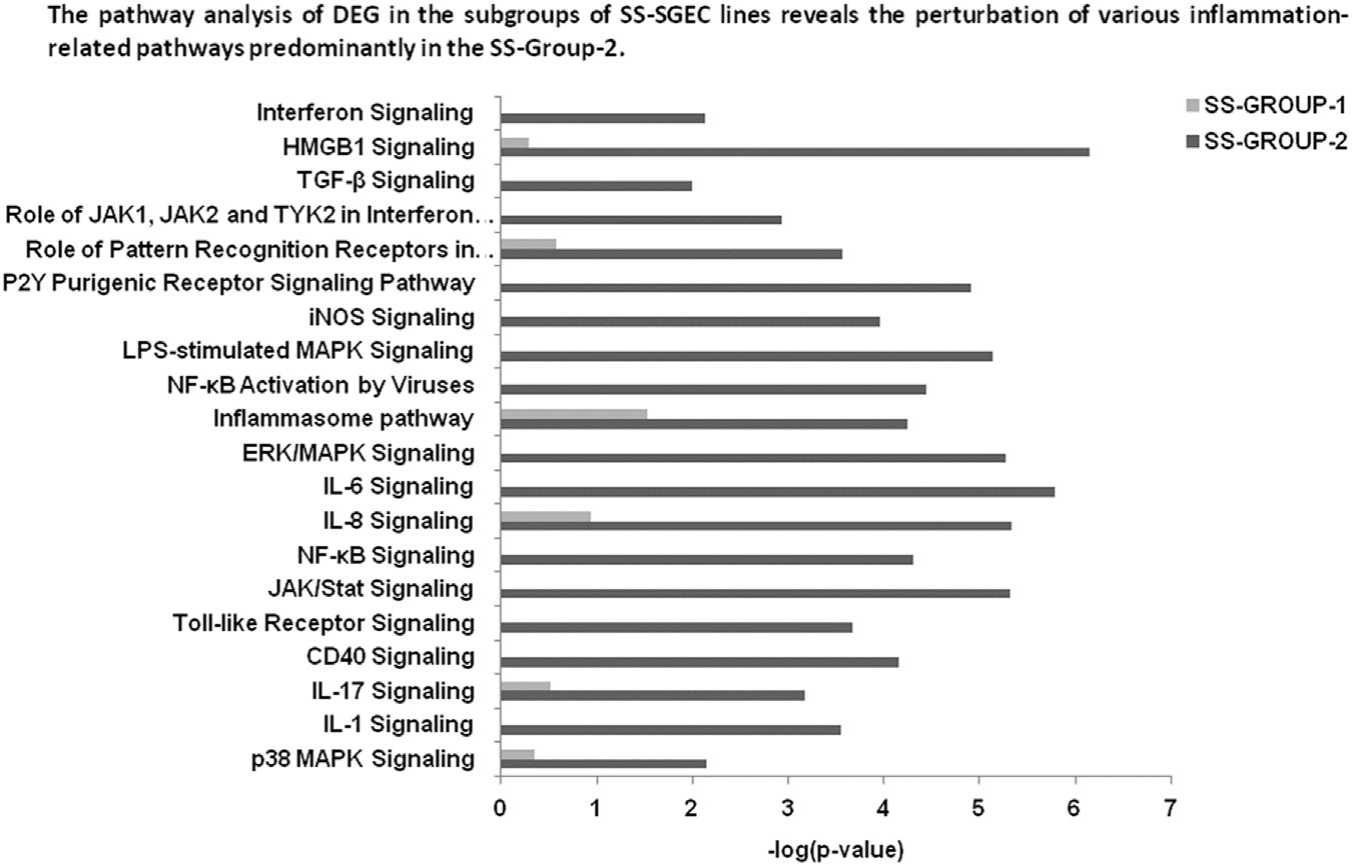
Perturbation of specific inflammation-related pathways in the SS-Group-2 of SS-SGEC lines compared to the SS-Group-1**.** The grouping of DEG into signaling pathways by ingenuity pathway analysis (IPA) in the SS-Group-1 and the SS-Group-2 of SS-SGEC lines studied is shown. Detailed presentations of genes that belong to each pathway for each of the two SS subgroups are provided in the respective Excel files 1 and 2.

**Fig. 5:**
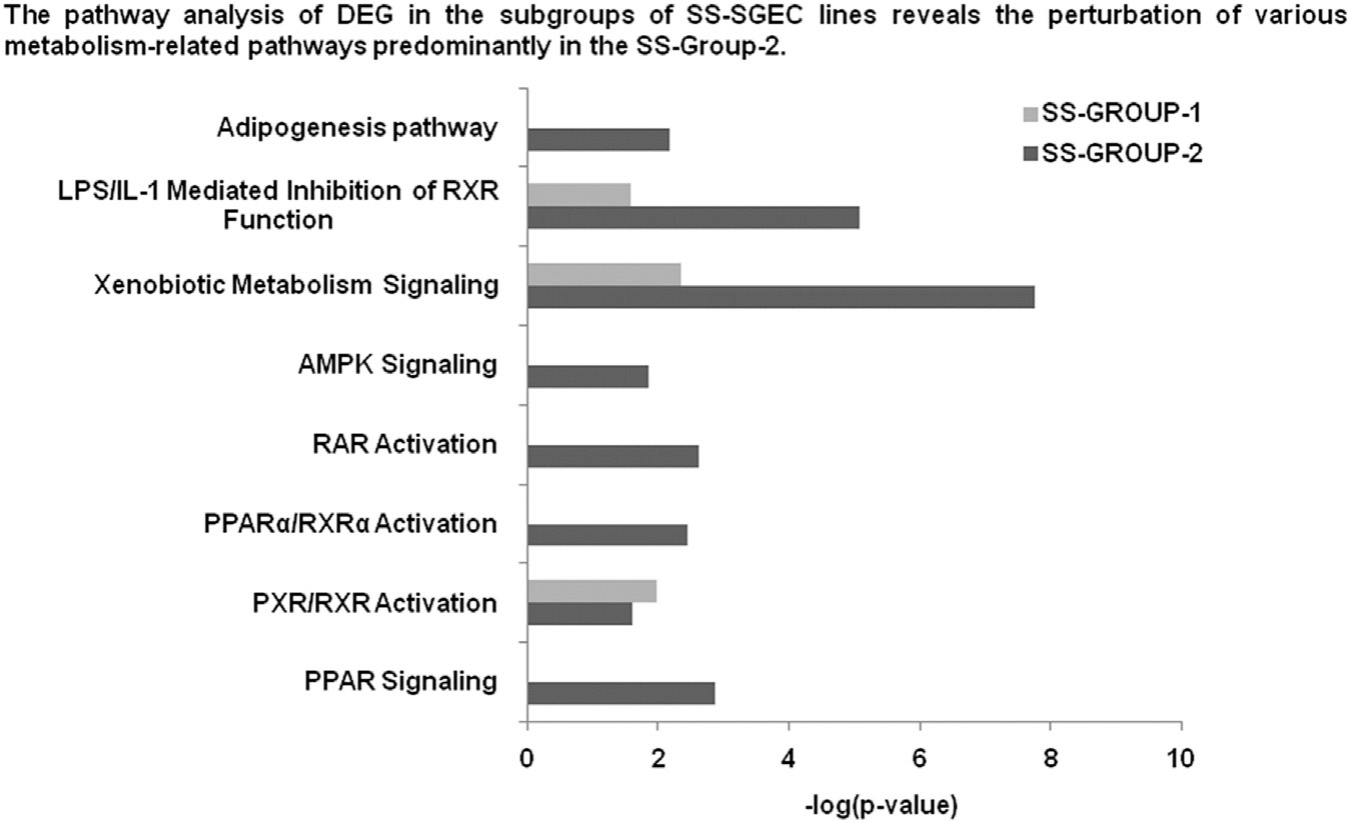
Perturbation of specific metabolism-related pathways in the SS-Group-2 of SS-SGEC lines compared to the SS-Group-1**.** Grouping of DEG into signaling pathways by ingenuity pathway analysis (IPA) in the SS-Group-1 and the SS-Group-2 of SS-SGEC lines studied is shown. Detailed presentations of genes that belong to each pathway for each of the two SS subgroups are provided in the respective Excel files 1 and 2.

The data presented here are related to the research article entitled **“***Impaired anti-inflammatory activity of PPARγ in the salivary epithelia of Sjögren's syndrome patients imposed by intrinsic NF-κB activation*” [1]. To better examine the hitherto gathered evidence of epithelial deregulation in SS patients [2], we performed for the first time comparative analysis of gene expression profiles in long-term cultured non-neoplastic SGEC lines derived from SS patients (SS-SGEC; *n* = 9) and non-SS controls (normal-SGEC lines; *n* = 3). The SS-SGEC lines studied included two subgroups, which were selected on the basis of the histological severity of lymphoepithelial infiltrations found in the MSG biopsies from which they were obtained (SS-Group-1; *n* = 3, focus score ≤ 2 and SS-Group-2; *n* = 6, focus score > 2). Fig. 1**A** and Excel File 1 present the differentially expressed genes (DEG), which were obtained following the comparisons between the various groups of SGEC lines studied. Selected DEG (3 up-regulated and one down-regulated) were validated by quantitative RT-PCR (Fig. 2). Fig. 1**B** and Fig. 3 show the hierarchical clustering of DEG and of total genes studied, respectively. The various signaling pathways found affected in the various groups of SS-SGEC lines are presented in Excel File 2. The analysis of the subgroups of SS-SGEC lines revealed the perturbation of various inflammation- and metabolism-related signaling pathways primarily in the SS-Group-2, including those of inflammasome, NF-κB, PPAR and pattern recognition receptors (Fig. 4, Fig. 5).

## 2 Experimental design, materials and methods

### 2.1 Patients and cell lines

Long-term cultures of non-neoplastic secondary salivary gland epithelial cell (SGEC) lines were established from minor salivary gland(MSG) biopsies and maintained in serum-free keratinocyte basal medium (KBM; Clonetics), as previously described [1]. The study was approved by the Ethics Committee of the School of Medicine, National University of Athens, Greece (protocol no. 5107). The exclusive epithelial nature and ductal epithelial origin of cultured SGEC lines was routinely verified by morphology, as well as by the uniform and consistent expression of epithelial specific markers and the absence of markers indicative of other types of cells. All our SS patients were diagnosed on the basis of the American–European SS classification criteria. The SS-SGEC lines studied were selected on the basis of the intensity of lymphoepithelial infiltrates in the respective MSG biopsies (all with sialadenitis focus score ≥ 1), and consisted of two subgroups; SS-Group-1 (*n* = 3) derived from biopsies with moderate lymphocytic infiltrations (focus score < 2) and SS-Group-2 (*n* = 6) derived from biopsies with severe infiltrations (focus score ≥ 2). Normal-SGEC lines, used as controls, were derived from non-SS individuals who had sicca complaints, but did not fulfill the SS classification criteria and did not have any histopathologic or serologic evidence for SS.

### 2.2 Reverse transcription–polymerase chain reaction (RT–PCR)

Total RNA was extracted with the RNeasy mini kit (Qiagen). RNA (0,25 μg) was reversed transcribed using High-Capacity RNA-to-cDNA™ Kit (Applied Biosystems®). Pro-IL1β, Versican, Pycard/ASC and NFKBIA mRNA were analyzed by quantitative Real-time PCR using commercially available primers specific for each gene (TaqMan® Gene Expression Assays; Applied Biosystems). All samples were run in duplicate. PCR program included an initial denaturation step at 95 °C for 10-min followed by 50 cycles of 95 °C for 15-s and by a final extension step at 60 °C for 1-min. Analysis was performed by the 2^–ΔΔCT^ method using HPRT1 as reference gene and HeLa cells as calibrator.

### 2.3 Microarray gene expression profiling

Transcriptome analyses were performed in total RNA specimens isolated from long-term cultured SGEC lines derived from non-SS controls (normal-SGEC lines; n = 3) and from SS patients (SS-SGEC; *n* = 9), using the Affymetrix microarray technology (GeneChipHuGene 1.0ST arrays with 28,869 annotated genes, following the manufacturer's workflow. Microarray analysis was performed with the R statistical environment version 2.13 using the Bioconductor package [3]. RMA normalization was performed in the microarray and identification of differentially expressed genes was conducted with Student's t-test (*p*-value < 0.05) [4]. Gene ontology annotation of differentially expressed genes, signal pathway analysis and network construction was performed with the use of Ingenuity Pathway Analysis (IPA) software and the Kyoto Encyclopedia of Genes and Genomes (KEGG). All microarray data are available in the GEO archive under the accession number GSE97614.

### 2.4 Statistical analyses

Correlations were calculated using the Spearman's rank correlation coefficient. Group comparisons were performed by Mann-Whitney rank sum test. For paired comparisons, Wilcoxon signed-rank test and one-way analysis of variance were used, when appropriate. Analyses were conducted using SPSS 15.0 and Graph Pad 5.0 softwares. Clustering of selected differentially expressed genes was performed with euclidean distance metric and average linkage with MultiExperiment Viewer software [5].

## Appendix A Supplementary information

Supplementary material **Excel file 1. Differentially expressed genes (DEG) in the various groups of SS-SGEC lines studied.** Excel file that displays the DEG observed following the various group comparisons (in separate Excel sheets) and include SS-Group-1 SGEC (n=3) versus normal-SGEC (n=3), SS-Group-2 SGEC (n=6) versus normal-SGEC (n=3), SS-Group-2 SGEC (n=6) versus SS-Group-1 SGEC (n=3), and total SS-SGEC lines (n=9) studied versus normal-SGEC (n=3). DEG include up-regulated genes with fold-change >1.5, and down-regulated genes with fold-change <0.67 (both for p<0.05). SS-Group-1 SGEC lines (M1-M3): derived from SG biopsies with focus score <2, SS-Group-2 of SS-SGEC lines (S1-S6): derived from SG biopsies with focus score ≥2.

Supplementary material **Excel file 2.Annotation of differentially expressed genes (DEG) to signaling pathways in the various groups of SS-SGEC lines studied.** Excel file that displays the canonical pathways that are enriched with DEG observed, as revealed by IPA. The various group comparisons are shown (in separate Excel sheets) and include SS-Group-1 SGEC (n=3) versus normal-SGEC (n=3), SS-Group-2 SGEC (n=6) versus normal-SGEC (n=3), SS-Group-2 SGEC (n=6) versus SS-Group-1 SGEC (n=3), and total SS-SGEC lines (n=9) studied versus normal-SGEC (n=3). The −log (p-value) indicates the minimum significance level derived from Fisher's exact test. The ratio refers to the number of genes from the dataset that map to the pathway enlisted divided by the total number of genes, which map to the canonical IPA pathway. SS-Group-1 SGEC lines (M1-M3): derived from SG biopsies with focus score <2, SS-Group-2 of SS-SGEC lines (S1-S6): derived from SG biopsies with focus score ≥2.
